# Multifunctional Cinnamic Acid Derivatives

**DOI:** 10.3390/molecules22081247

**Published:** 2017-07-25

**Authors:** Aikaterini Peperidou, Eleni Pontiki, Dimitra Hadjipavlou-Litina, Efstathia Voulgari, Konstantinos Avgoustakis

**Affiliations:** 1 Department of Pharmaceutical Chemistry, School of Pharmacy, Faculty of Health Sciences, Aristotle University of Thessaloniki, Thessaloniki 54124, Greece; katerina.peperidou@gmail.com (A.P.); epontiki@pharm.auth.gr (E.P.); 2 Department of Pharmaceutical Technology and Pharmaceutical Analysis, School of Pharmacy, University of Patras, Rio Patras 26504, Greece; efiv48@hotmail.com (E.V.); avgoust@upatras.gr (K.A.)

**Keywords:** cinnamic acids, multitarget, lipoxygenase inhibitors, antiproteolytic activity

## Abstract

Our research to discover potential new multitarget agents led to the synthesis of 10 novel derivatives of cinnamic acids and propranolol, atenolol, 1-adamantanol, naphth-1-ol, and (benzylamino) ethan-1-ol. The synthesized molecules were evaluated as trypsin, lipoxygenase and lipid peroxidation inhibitors and for their cytotoxicity. Compound **2b** derived from phenoxyphenyl cinnamic acid and propranolol showed the highest lipoxygenase (LOX) inhibition (IC_50_ = 6 μΜ) and antiproteolytic activity (IC_50_ = 0.425 μΜ). The conjugate **1a** of simple cinnamic acid with propranolol showed the higher antiproteolytic activity (IC_50_ = 0.315 μΜ) and good LOX inhibitory activity (IC_50_ = 66 μΜ). Compounds **3a** and **3b**, derived from methoxylated caffeic acid present a promising combination of in vitro inhibitory and antioxidative activities. The *S* isomer of **2b** also presented an interesting multitarget biological profile in vitro. Molecular docking studies point to the fact that the theoretical results for LOX-inhibitor binding are identical to those from preliminary in vitro study.

## 1. Introduction

The biological interest of cinnamic acids (CAs), ferulic, caffeic and other phenolic CAs has been identified by many research groups and attributed to their anti-inflammatory [1,2,3], anti-oxidative [4], anti-tumor [5], anti-microbial [6], multiple cytoprotective actions ameliorating neuro inflammation in neurogenerative diseases [7], anti-hypertensive and anti-hyperlipidemic activities minimizing the oxidation of low-density lipoprotein (LDL) [8].

Cinnamic acid significantly reduces the body weight of obese rats and inhibits angiotensin converting enzyme (ACE) activity in serum [9]. In addition, it improves vasoconstriction and hypertension complications presenting a cardioprotective profile [9].

Thus, acknowledging CA derivatives’ multiple and high efficacy, their potential for improving human health either as a single molecule and/or in combination with other drugs in the form of hybrids seems to be broad. Hybrid drugs combine two drug entities in a single molecule, capable of interacting simultaneously with multiple targets, directly or following metabolism supporting the philosophy of polypharmacology [10,11].

Polypharmacology, which is emerging as the new paradigm of drug discovery [12,13,14,15,16] includes: (a) single drugs acting on multiple targets of a unique disease pathway, or (b) single drugs acting on multiple targets pertaining to multiple disease pathways.

Recently, combinations of appropriate pharmacophores with cinnamic acid derivatives have been developed to identify promising drug candidates as inhibitors of multiple biological targets associated with inflammation [17,18]. Phenolic conjugates combining the gallic acid and caffeic acid scaffolds [19,20] can act as potential antioxidants preventing the abnormal oxidative status linked to neurodegenerative, inflammatory or cancer processes. CA derivatives containing different amino acids [21] show a prolonged (up to 54 h) duration of action in the heart system. They are beneficial for cardiomyopathy and help to retain normal cardiac function.

During the last decade, our group has designed and synthesized several cinnamic acid derivatives as potent lipoxygenase inhibitors, antioxidant, anti-cancer and anti-inflammatory agents [2,3,22,23]. In continuation of our previously reported work [24], we have now used these particular CAs in order to design and synthesize two series of novel cinnamic acid-based agents as pleiotropic candidates against multiple inflammation targets. These new derivatives can be divided into two categories: (a) esters of cinnamic acid; and (b) amides of cinnamic acid, combined with: (i) known β-blockers drugs such as propranolol and atenolol; and (ii) drug-like molecules, e.g., 1-adamantanol, naphthalen-1-ol and 2-(benzylamino)-ethan-1-ol.

We envisaged integrating in the same molecule antioxidant, anti-lipoxygenase (anti-LOX) and anti-proteolytic activities to obtain compounds with multitarget responses. Another objective of the present study was to evaluate the effect of steric and electronic parameters on anti-LOX activity and to optimize the activity through systematic modification of the substituents on the phenylacrylic acid core. The new compounds were to be evaluated for their: (a) antioxidant activity; (b) ability to inhibit soybean lipoxygenase; and (c) ability to inhibit trypsin. To interpret the in vitro results representative derivatives would be further subjected to modeling studies.

## 2. Results and Discussion

### 2.1. Chemistry

**Scheme 1:**
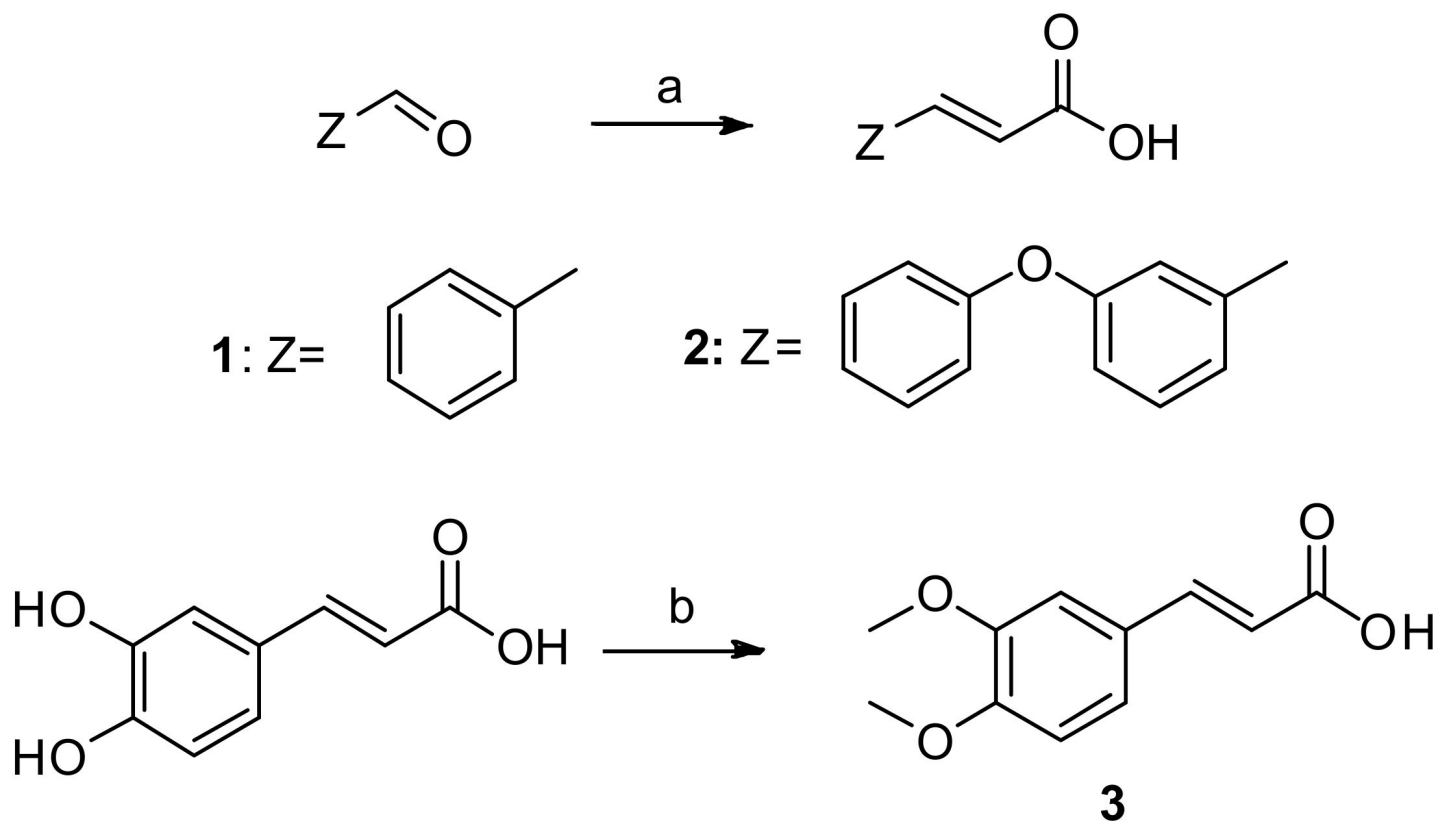
Synthesis of cinnamic acids. Reagents and reaction conditions: (**a**) pyridine; CH_2_(COOH)_2_; piperidine; reflux; 120–140 °C; 4 h; (**b**) acetone, 4 M aqueous solution of NaOH; reflux 4 h.

**Scheme 2:**
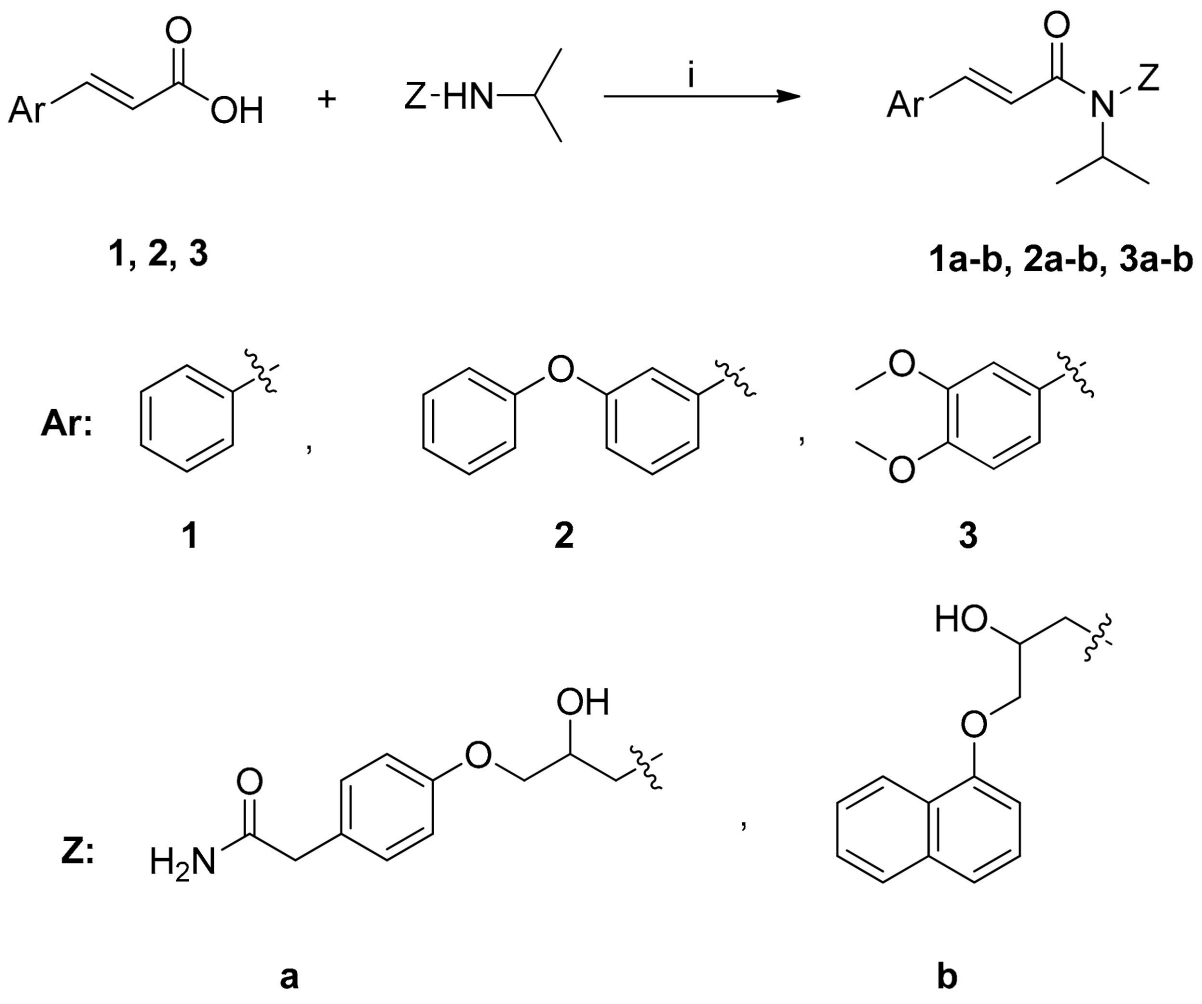
Synthesis of cinnamic amides. Reagents and reaction conditions: (i) dry CH_2_Cl_2_/Et_3_N; BOP-reagent; stirring at r.t. for 24 h.

**Scheme 3:**
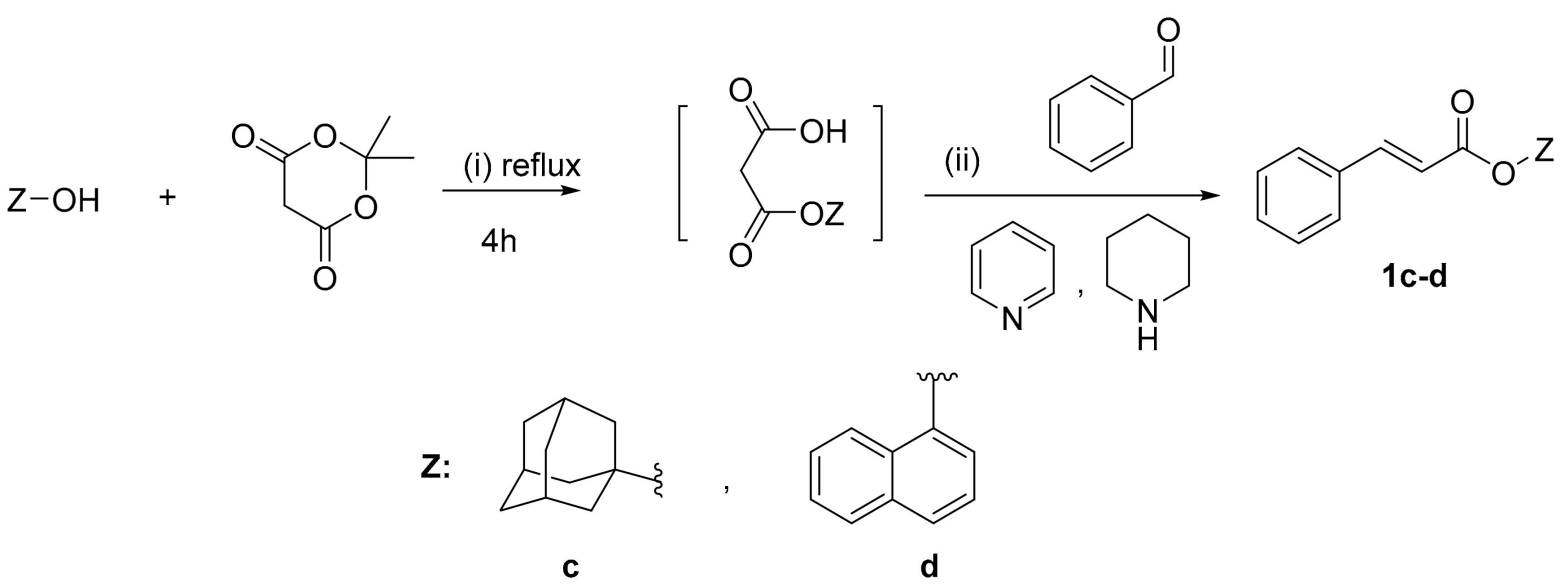
Synthesis of cinnamic esters **1c**–**d**. Reagents and reaction conditions: (i) Mono-esterification: Meldrum acid, toluene, 4 h reflux; (ii) Condensation: pyridine, piperidine, stirring at r.t. for 24 h.

**Scheme 4:**
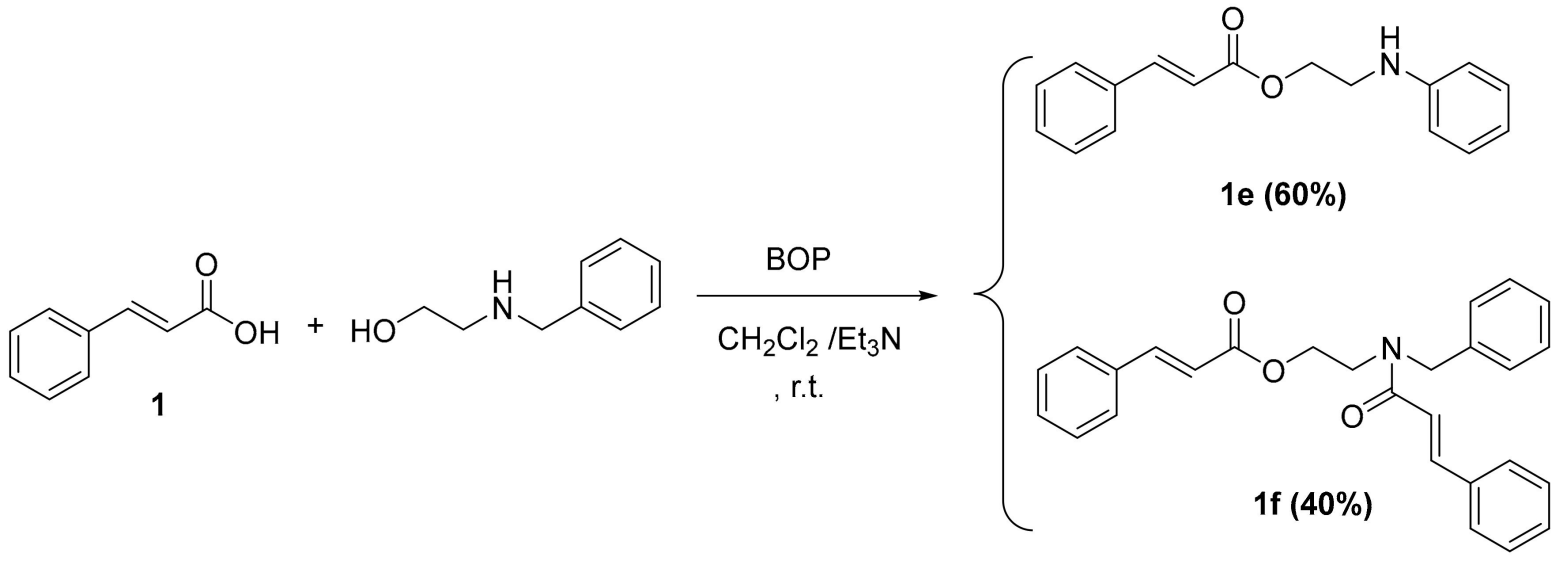
Synthesis of cinnamic derivatives **1e**–**f**. Reagents and reaction conditions: dry CH_2_Cl_2_/Et_3_N; BOP-reagent; stirring at r.t. for 24 h.

The design of the new multitarget cinnamic derivatives was based on our previous research [24] combining three moieties: the enoyl-acyl backbone part, drugs and the drug-like molecules attached to the functionality of the CAs (Scheme 1, Scheme 2, Scheme 3 and Scheme 4) following Lipinski's rules. Variations were accomplished with the choice of suitable substituted cinnamic acids which showed interesting biological behavior by themselves, drugs and drug-like molecules.

The synthesis of cinnamic acids **1**, **2** was established by a Knoevenagel–Doebner condensation of a suitable aldehyde with malonic acid in the presence of pyridine and piperidine, as shown in Scheme 1 [1,24]. The synthesis of 3,4-dimethoxycaffeic acid (**3**) was based on the methylation of caffeic acid using Me_2_SO_4_.The physicochemical and spectroscopic data of the obtained acids were identical to those given in the literature.

The synthesis of cinnamic amides **1a**–**b**, **2a**–**b**, **3a**–**b** proceeded through the reaction of the appropriate cinnamic acid and the suitable known β-blocker drugs propranolol and atenolol, respectively (Scheme 2). The synthesis of these amides followed an one pot procedure in anhydrous dichloromethane (DCM) using *O*-(benzotriazol-1-yl)-*N*,*N*,*N*′,*N*″-tetramethyluronium hexafluoro-phosphate (BOP) (Scheme 2) in the presence of triethylamine as a base.

The synthesis of cinnamic ester derivatives **1c**–**f**, proceeded through the reaction of the cinnamic acid and 1-adamantol, naphthalen-1-ol or 2-(benzylamino) ethan-1-ol, respectively, based on different conditions. Specifically, the synthesis of cinnamic esters **1c**–**d** was actually based on a condensation reaction using the Meldrum acid reagent under basic conditions, as it is reported in the literature [25]. The synthesis of cinnamic esters **1e**–**f** was based on one pot reaction using BOP as a coupling reagent [24,26].

The final products were obtained in good yields (50–92%), with the only exception of compound **2c** which was obtained in lower yield (32%). The pure final products were recrystallized from appropriate solvents or subjected to preparative thin layer chromatography (prep TLC). IR, ^1^H-NMR, ^13^C-NMR and elemental analysis were used to confirm the structures of the synthesized compounds. All the esters and amides presented characteristic IR absorptions (nujol or KBr disk) at 1720 (C=O), and 1625 (C=C), cm^−1^) and correspond to the *E*-isomers (*J* > 9 Hz). The ^1^H-NMR and ^13^C-NMR data confirmed the proposed structures. The LC-MS (ESI) examination showed: [Μ + 1]^+^ as well as [Μ + 1 + Νa]^+^, [Μ + 1 + Κ]^+^, [Μ + 1 + Νa + ΜeOH]^+^ peaks.

### 2.2. Physicochemical Studies

Since lipophilicity is a significant physicochemical property determining distribution, bioavailability, metabolic activity in and elimination from the human body, we tried to determine experimentally the lipophilicity of the synthesized hybrids using the RPTLC method as *R*_M_ values [27]. This is considered to be a reliable, fast and convenient method for expressing lipophilicity (Table 1). *R*_M_ refers to the thermodynamic *R*_f_ ‘value in a logarithmic form. This so-called *R*_M_ value was introduced in 1950 by Bate-Smith and Westall [28]. Amide **1b** (0.411), which is a combination with propranolol, is more lipophilic than the parent acid **1** (−0.485) as well as more lipophilic than the amide **1a** of atenolol (−0.720). The individual structural characteristics explain these differences. Perusal of *R*_M_ values indicates that amide **2b** presents higher lipophilicity than **2a**: **2b** > **2a** > **2**. Again, the propranolol hybrid is more lipophilic. Its special structural characteristics e.g., the naphthyl group supports the result. The same results are given by the amides of acid **3** (**3b** > **3a** > **3**). Esters **1c**–**f** are more lipophilic than the corresponding acid **1** (**1c** > **1d** > **1f** > **1e**).

### 2.3. Biological Evaluation

In the present investigation, the new CA derivatives as well as the parent molecules were studied with regard to their antioxidant ability as well as to their ability to inhibit soybean LOX in comparison with well-known antioxidant agents recommended as references, e.g., nordihydroguaiaretic acid (NDGA) and Trolox. In addition, they were tested as anti-proteolytic agents.

Over the past decade, the study of the biological activity and significance of Reactive Oxygen Species (ROS) have gained particular interest from both a physiological and pathological perspective. Since ROS are highly unstable and reactive molecules, they interfere with many cellular processes. ROS react with lipids, nucleic acids and proteins, disrupting their cellular functions. Oxidative stress occurs when the damaging effects of ROS exceed the ability of biological systems to neutralize the oxidizing agents and to repair cellular damage. Oxidative stress and chronic low-grade inflammation are interdependent processes that have been implicated in aging and many pathological conditions like cardiovascular diseases, neurodegenerative diseases or cancer. Inflammatory cells can release ROS at the site of inflammation increasing oxidative stress, while ROS can initiate intracellular signaling cascades that increase pro-inflammatory gene expression [29,30]. Physiologically, antioxidant defenses are efficient enough to neutralize the damaging effect of oxidizing molecules [31,32,33].

Antioxidants in generally, even at low concentration, significantly delay or prevent oxidation of easily oxidizable substrates. Biological systems protect themselves against the damaging effects of activated species by several free radical scavengers, enzymes and chain reaction [34].

It is well established that many non-steroidal anti-inflammatory drugs have been reported to act either as inhibitors of free radical production or as radical scavengers [35]. Antioxidants acting as lipid peroxidation inhibitors could be beneficial for health’s maintenance and for the compensation of risk factors [36]. Thus, we tested the present conjugates with regard to their antioxidant ability.

In our studies, 2,2-azobis (2-amidinopropane) dihydrochloride (AAPH) was used as a free radical initiator to follow oxidative changes of linoleic acid to conjugated diene hydro peroxide. Azo compounds generating free radicals through spontaneous thermal decomposition are useful for free radical production studies in vitro [36]. The water-soluble azo compound AAPH has been extensively used as a clean and controllable source of thermally produced alkylperoxyl free radicals. The activity of the peroxyl radicals produced by the action of AAPH shows a greater similarity to cellular activities such as lipid peroxidation [36]. In the AAPH assay, the highly reactive alkylperoxyl radicals are intercepted mainly by a hydrogen atom transfer (HAT) from the antioxidant [37]. Therefore, particularly effective HAT agents are compounds with high hydrogen atom donating ability, that is compounds with low heteroatom-H bond dissociation energies and/or compounds from which hydrogen abstraction leads to sterically hindered radicals as well as compounds from which abstraction of hydrogen leads to C-centered radicals stabilized by resonance.

Cinnamic acids **1**–**3** were found to be quite potent lipid peroxidation inhibitors (78–86%). Hybrid amides **1a**–**b**, **2a**–**b** and **3a**–**b**, all exhibited significant anti-lipid peroxidation activity. Within the group of esters **1d** and **1f** seemed inactive under the experimental conditions used, while **1c** and **1e** presented moderate activity. The presence of the cinnamoyl group is correlated with the activity which is partly influenced by the structural modifications of substituents Ar and Z.

Eicosanoids are oxygenated metabolites of arachidonic acid with implication in a broad diversity of diseases. Upon appropriate stimulation of neutrophils, arachidonic acid (AA) is cleaved from membrane phospholipids and can be converted into leukotrienes (LTs) by LOXs [38]. Leukotriene B_4_ (LTB_4_) generation is considered to be important in the pathogenesis of neutrophil-mediated inflammatory diseases [39] with a marked relation to the severity of cardiovascular diseases, asthma and cancer. Previously published results suggest that cardiac 12/15-LOX is involved in the development of heart failure and that inhibition of 12/15-LOX could be a novel treatment for this condition [40]. The cinnamic hybrids were therefore expected to offer inhibition of LOX since their CA precursors are potent LOX inhibitors. The synthesized derivatives were tested in vitro against soybean lipoxygenase (LOX) by the UV-based enzyme assay [24]. For the sake of comparison atenolol and propranolol, the starting materials, were tested too. Study of LOX IC_50_ inhibition values demonstrates that amide **2b** is by far the most active inhibitor, followed by compounds **3a** and **3b** (Table 1) as well hybrids **2a** > **1b** > **1a**. It seems that in general these molecules are much more potent compared to their precursors acids (especially acid **3** in which the catechol hydroxyl groups were methylated) and more potent than atenolol and propranolol. Esters **1c**, **1d**, **1e** and **1f** exhibit low or no activity.

Lipophilicity is referred to as an important physicochemical property for both LOX inhibition and lipid peroxidation inhibition [41]. The most potent compound **2b** seems to follow this concept (*R*_M_ value 0.907). However, regression analyses of the values of IC_50_ revealed that the lipophilicity of the molecules expressed as *R*_M_ values (experimental) does not govern the biological response. For instance, the highly lipophilic compound **1c** (0.911) seemed inactive, whereas compounds **3a** and **3b** with *R*_M_ values −0.841 and 0.0819 (Table 1) are very potent. Compounds **3a** and **3b** are equipotent (IC_50_ = 10 µM). They all are derivatives of the same acid **3**. Considering the structural characteristics, it seems that the amides are more potent inhibitors than the esters. Most of the LOX inhibitors are antioxidants or free radical scavengers [42]. LOXs contain a “non-home” iron per molecule in the enzyme active site as high-spin Fe^2+^ in the native state and the high spin Fe^3+^ in the activated state. Some studies suggest a relationship between LOX inhibition and the ability of the inhibitors to reduce Fe^3+^ at the active site to the catalytically inactive Fe^2+^. This inhibition is related to their ability to reduce the iron species in the active site to the catalytically inactive ferrous form [38], whereas several LOX inhibitors are excellent ligands for Fe^3+^. NDGA, a known inhibitor of soybean LOX, has been used as a reference compound (IC_50_ 0.45 μΜ) and as a positive control. Herein it seems that LOX inhibition is correlated with the anti-lipid peroxidation activity, with some exceptions (**1d** and **1f**).

Serine proteases are also implicated in the pathophysiology of inflammatory diseases. Host defense reactions play an important role in the propagation of the disease states exacerbating the local events and ultimately leading to tissue damage. We evaluated the ability of our compounds as well as of their precursors (CAs, atenolol and propranolol) to inhibit trypsin, a well-known protease. **1a** was a highly potent inhibitor (IC_50_ = 0.315 μM) followed by compounds **2a**, **2b**, **3b**, **3a** and **1b**. The esters did not present any or low anti-proteolytic activity. In general, all the amides exhibit anti-proteolytic activity higher than the corresponding acids (only acid **1** presents inhibitory activity). Atenolol and propranolol did not present any activity under our experimental conditions. Again, the amides were more potent. Conjugation of atenolol with CA **1** or **2** is correlated with higher activity (**1a** and **2a**). It is interesting that the *S* isomer of **2b** significantly combines the anti-LOX (6 μM) and anti-proteolytic activity (2.75 μM).

**Figure 1:**
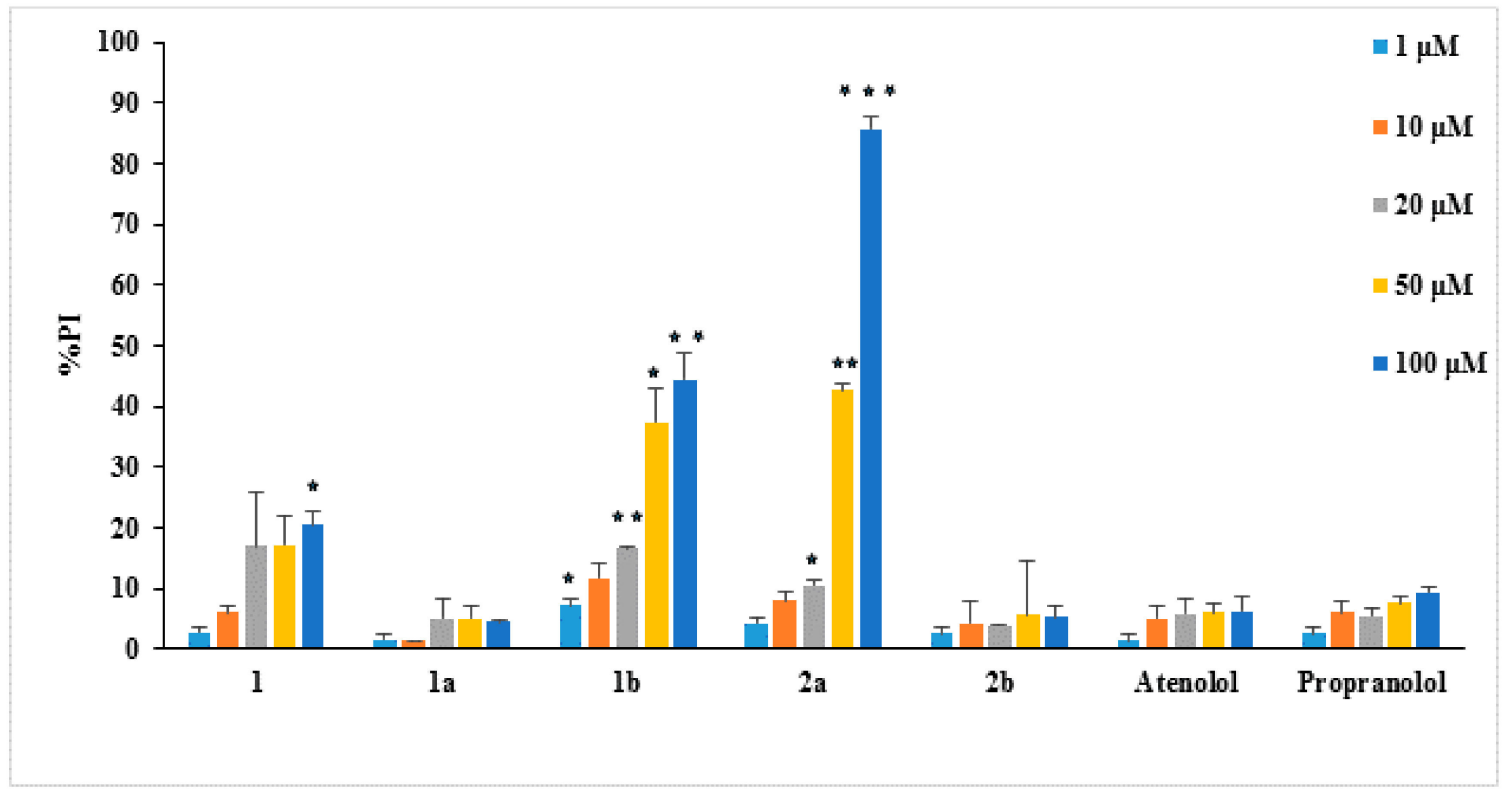
Cytotoxicity of compounds on L929 cells (24 h incubation), as PI% values. The lines on the bars indicate standard deviation. Asterisks indicate statistical significance of difference between the respective compound and atenolol at the respective concentration (* *p* < 0.05, ** *p* < 0.01, *** *p* < 0.001).

The cytotoxicity of the synthesized derivatives was determined using the propidium iodide (PI) fluorescence method [43] in the presence of different concentrations (1–100 μM) of these compounds. L929 mouse fibroblasts cells were used in this work since they have been previously used in the study of the pharmacological effects of antioxidant and anti-inflammatory agents [44,45]. The cytotoxicity results of the new compounds against normal cells (such as the L929 fibroblasts) would give useful information as only non-cytotoxic compounds should be further evaluated as potential antioxidant drugs. The results are presented in Figure 1 in the form of the % cell survival values as propidium iodide % (PI)—values PI% for the examined compounds. Among the tested compounds only **1b** and **2a** presented remarkable cytotoxicity.

### 2.4. Computational Studies—Docking Simulations on Soybean Lipoxygenase

**Figure 2:**
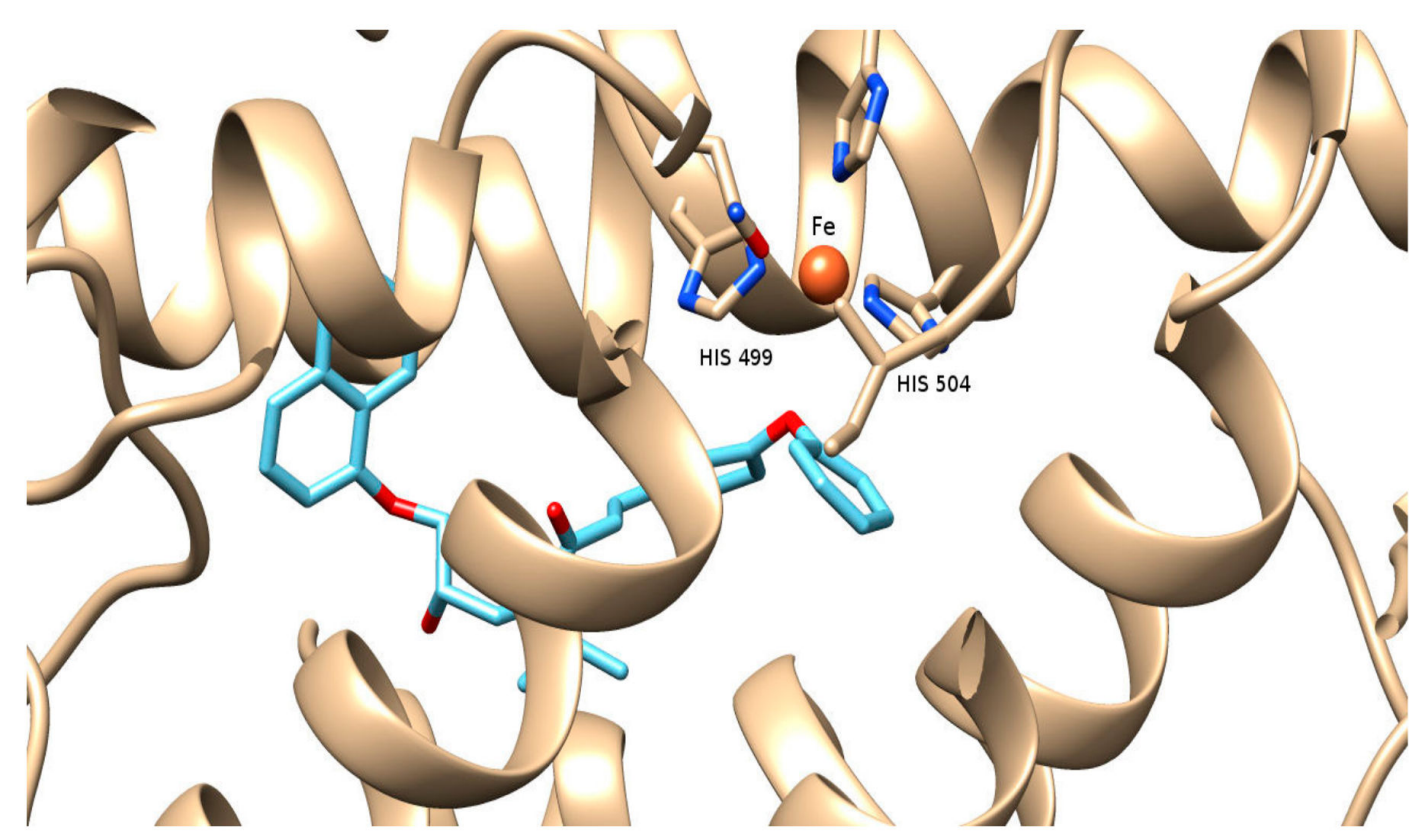
Docking pose of **2b** (depicted in blue and red) bound to soybean lipoxygenase (LOX-1). The iron ion is depicted as an orange sphere.

The molecular modeling study performed provided useful interpretation of the experimental results. The binding of **2b** to soybean LOX (PDB code: 3PZW) has a higher AutoDock Vina score than any of the other derivatives docked. The preferred docking orientation for the most potent derivative **2b** is shown in Figure 2. The oxygen of the diphenyl ether of **2b** could coordinate with the iron of the active site. Furthermore, **2b** is able to accommodate the extensively hydrophobic cavity close to the active site, incorporating His 504 and His 499 among other residues with possible hydrophobic interactions (π-π stacking). It is likely that the extension scaffold of **2b** into the hydrophobic domain blocks approach of substrate to the active site and hence prevents oxidation by soybean LOX.

## 3. Experimental Section

### 3.1. General Information

All chemicals, solvents, chemical and biochemical reagents were of analytical grade and purchased from commercial sources (Merck, Merck KGaA, Darmstadt, Germany, Fluka Sigma-Aldrich Laborchemikalien GmbH, Hannover, Germany, Alfa Aesar, Karlsruhe, Germany and Sigma, St. Louis, MO, USA). Soybean lipoxygenase, pancreatic bovine trypsin, sodium linoleate, 2,2-azobis (2-amidinopropane) dihydrochloride (AAPH) were obtained from Sigma Chemical, Co. (St. Louis, MO, USA). All starting materials were obtained from commercial sources (Merck, Merck KGaA, Darmstadt, Germany, Fluka Sigma-Aldrich Laborchemikalien GmbH, Hannover, Germany, Alfa Aesar, Karlsruhe, Germany and Sigma, St. Louis, MO, USA) and used without further purification.

Melting points (uncorrected) were determined on a MEL-Temp II (Lab. Devices, Holliston, MA, USA). For the in vitro tests, UV-Vis spectra were obtained on a 554 double beam spectrophotometer Perkin-Elmer (Perkin-Elmer Corporation Ltd., Lane Beaconsfield, Bucks, UK). Infrared spectra (film as Nujol mulls or KBr pellets) were recorded with Perkin-Elmer 597 spectrophotometer (Perkin-Elmer Corporation Ltd., Lane Beaconsfield, Bucks, England). The ^1^H Nucleic Magnetic Resonance (NMR) spectra were recorded at 300 MHz on a Bruker AM-300 spectrometer (Bruker Analytische Messtechnik GmbH, Rheinstetten, Germany) in CDCl_3_ or DMSO using tetramethylsilane as an internal standard unless otherwise stated. ^13^C-NMR spectra were obtained at 75.5 MHz on a Bruker AM-300 spectrometer in CDCl_3_ or DMSO solutions with tetramethylsilane as internal reference unless otherwise stated. Chemical shifts are expressed in δ (ppm) and coupling constants *J* in Hz. Mass spectra were determined on a LC-MS 2010 EV Shimadzu (Shimadzu, Kiyoto, Japan) using MeOH as solvent. Elemental analyses for C and H gave values acceptably close to the theoretical values (±0.4%) in a Perkin-Elmer 240B CHN analyzer (Perkin-Elmer Corporation Ltd., Lane Beaconsfield, Bucks, UK). Reactions were monitored by thin layer chromatography on 5554 F_254_ Silica gel/TLC cards (Merck and Fluka Chemie GmbH Buchs, Steinheim, Switzerland). For preparative thin layer chromatography (prep TLC) Silica gel 60 F_254_, plates 2 mm, Merck KGaA ICH078057 were used. For the experimental determination of the lipophilicity using reverse phase thin layer chromatography (RP-TLC) TLC-Silica gel 60 F_254_ DC Kieselgel, Merck (20 × 20 cm) plates were used.

### 3.2. Chemistry General Procedure

#### 3.2.1. General Procedure for One Pot Synthesis of Cinnamic Amides **1a**–**b**, **2a**–**b** and **3a**–**b**

To a stirred solution of the appropriate cinnamic acid **1**, **2** or **3** (1 equivalent) and propranolol or atenolol (1.1 equivalents) in dichloromethane (25 mL), BOP (1.2 equivalents) and triethylamine (4 equivalents) were added [24,26]. The end of the reaction was monitored by TLC. After 24 h of stirring at room temperature, the dichloromethane was removed under reduced pressure and ethyl acetate (80 mL) was added to the residue. The resulted solution was washed with 10% citric acid (2 × 20 mL), 10% solution of NaHCO_3_ (3 × 20 mL), water (20 mL) and brine (2 × 20 mL), dried over anhydrous sodium sulfate, filtered and concentrated under reduced pressure. The crude product was purified by preparative thin layer chromatography (Prep TLC) or crystallized from the appropriate solvent or triturated from diethyl ether.

*(E)-N-(3-(3-(2-amino-2-oxoethyl) phenoxy)-2-hydroxypropyl)-N-isopropylcinnamamide* (**1a**). The crude product was purified by prep TLC (ethyl acetate/petroleum ether 2:1). Yield: 92%; *R*_f_ (petroleum ether:ethyl acetate 1:1): 0.2; m.p.: 102–105 °C; IR (KBr, cm^−1^): 3383.1, 3207.5, 1734.2, 1666.1, 1641.5; LC-MS (*m*/*z*): (C_23_H_28_N_2_O_4_) [M + 1] = 397, [M + Na^+^] = 419; ^1^H-NMR (CDCl_3_, δ): 1.29 (m, 6H, -CH_3_), 2.17 (d, 1H, aliphatic proton), 2.48 (bs, 1H, -OH), 3.54 (m, 2H, -NH_2_), 3.72 (m, 1H, aliphatic proton), 3.87 (m, 1H, aliphatic proton), 4.09 (m, 2H, aliphatic proton), 4.35 (m, 1H, aliphatic proton), 5.54 (m, 1H, aliphatic proton), 6.91 (m, 3H, aromatic), 7.20 (m, 2H, aromatic), 7.38 (m, 3H, aromatic), 7.53 (m, 2H, aromatic), 7.73 (dd, 1H, *J* = 15.2 Hz); ^13^C-NMR (CDCl_3_, δ): 19.51, 41.05, 43.51, 51.2, 68.12, 70.63, 113.89, 114.01, 118.53, 122.95, 128.41, 128.85, 129.51, 129.77, 135.33, 135.37, 142.80, 157.92, 167.86, 172.60; Elemental Analysis: Expected % (C_23_H_28_N_2_O_4_): C, 69.68; H, 7.12; N, 7.07; Found % (C_25_H_27_NO_3_): C, 69.83; H, 7.12; N, 7.05.

*(E)-N-(2-hydroxy-3-(naphthalen-1-yloxy) propyl)-N-isopropylcinnamamide* (**1b**). The crude product was crystallized from ethyl acetate Yield: 60%; *R*_f_ (petroleum ether:ethyl acetate 1:1): 0.4; m.p.: 158–160 °C; IR (KBr, cm^−1^): 3239.7, 2927.8, 1641.6, 1595.5; LC-MS (*m*/*z*): (C_31_H_31_NO_4_) [M + 1] = 390, [M + Na^+^] = 412, [M + Na^+^ + MeOH] = 444; ^1^H-NMR (CDCl_3_, δ): 1.30 (d, 3H, *J* = 6.5 Hz, -CH_3_), 1.42 (d, 3H, *J* = 6.5 Hz, -CH_3_), 3.68 (d, 1H, *J* = 14.6 Hz), 3.88 (dd, 1H, *J*_1_ = 14.6 Hz, *J*_2_ = 8.1 Hz), 4.09 (t, 1H, *J* = 9.9 Hz), 4.29 (d, 2H, *J* = 6.5 Hz), 4.43–4.39 (m, 1H, aliphatic), 6.95–6.92 (dd, 2H, *J*_1_ = 24.9 Hz, *J*_2_ = 11.4 Hz), 7.40 (m, 4H, aromatic), 7.45–7.52 (m, 3H, aromatic), 7.55–7.56 (m ,2H, aromatic), 7.75–7.82 (dd, 2H, *J*_1_ = 24.9 Hz, *J*_2_ = 11.4 Hz), 8.23–8.24 (d, 1H, *J* = 8 Hz); ^13^C-NMR (CDCl_3_, δ): 20.06, 43.27, 52.74, 68.33, 73.16, 106.95, 119.24, 119.37, 121.59, 121.87, 123.34, 124.09, 124.46, 126.02, 126.36, 126.65, 128.3, 130.06, 130.75, 133.95, 139.31, 140.54, 155.17, 156.43, 157.16, 168.33. Elemental Analysis: Expected % (C_25_H_27_NO_3_): C, 77.09; H, 6.99; N, 3.60; Found % (C_25_H_27_NO_3_): C, 76.79; H, 7.06; N, 3.78.

*(E)N-(3-(3-(2-amino-2-oxoethyl) phenoxy)-2-hydroxypropyl)-N-isopropyl-3-(3-phenoxy phenyl) acrylamide* (**2a**). The crude product was purified by Prep TLC (ethyl acetate/petroleum ether 2:1) and triturated from diethyl ether. Yield: 78%; *R*_f_ (petroleum ether:ethyl acetate 1:1): 0.21; m.p.: 131–133 °C; IR (KBr, cm^−1^): 3398.3, 3210.8, 2367.7, 1740.2, 1664.4, 1640.4; LC-MS (*m*/*z*): (C_29_H_32_N_2_O_5_) [M + 1] = 489, [M + Na^+^] = 511, [M + Na^+^ + MeOH] = 543; ^1^H-NMR (CDCl_3_, δ): 1.27 (d, 3H, *J* = 6.5 Hz, -CH_3_), 1.30 (d, 3H, *J* = 6.5 Hz, -CH_3_), 2.56–2.58 (m, 1H, aliphatic), 3.36–3.43 (m, 2H, aliphatic), 3.62–3.70 (m, 2H, aliphatic), 3.87–3.91 (m, 1H, aliphatic proton), 4.02–4.04 (m, 1H, aliphatic proton), 4.44–4.47 (m, 1H, aliphatic), 5.71 (m, 1H, aliphatic proton), 6.86–6.83 (d, 1H, *J* = 15 Hz), 6.90–6.92 (d, 3H, *J* = 8 Hz), 7.02 (d, 3H, *J* = 8 Hz), 7.14 (m, 1H, *J* = aromatic) 7.20 (s, 3H, aromatic), 7.35 (m ,3H, aromatic), 7.66–7.69 (d, 1H, *J* = 15 Hz); ^13^C-NMR (CDCl_3_, δ): 20.26, 41.11, 44.05, 52.36, 67.57, 68.69, 114.01, 114.29, 117.01, 118.24, 119.41, 120.36, 121.78, 122.94, 124.49, 130.85, 131.95, 131.98, 135.36, 139.14, 142.57, 154.85, 156.01, 158.2, 168.82, 171.58; Elemental Analysis: Expected % (C_29_H_32_N_2_O_5_): C, 71.29; H, 6.60; N, 5.73; Found % (C_29_H_32_N_2_O_5_): C, 71.17; H, 6.46; N, 5.58.

*(E)N-(2-hydroxy-3-(naphthalen-1-yloxy) propyl)-N-isopropyl-3-(3phenoxyphenyl) acrylamide* (**2b**). The crude product was purified by Prep TLC (ethyl acetate/petroleum ether 4:1).Yield: 79%; *R*_f_ (petroleum ether:ethyl acetate 1:1): 0.77; m.p.: 118–120 °C; IR (KBr, cm^−1^): 3254.9, 2980.9, 1702, 1675.9, 1637.7; LC-MS (*m*/*z*): (C_31_H_31_NO_4_) [M + 1] = 482, [M + Na^+^] = 504, [M + Na^+^ + MeOH] = 536, [M + K^+^] = 520; ^1^H-NMR (CDCl_3_, δ): 1.28–1.29 (d, 3H, *J* = 6.5 Hz, -CH_3_), 1.40–1.41 (d, 3H, *J* = 6.5 Hz, -CH_3_), 3.46–3.61 (m, 2H, aliphatic), 3.65–3.68 (m, 1H, aliphatic), 3.84–3.89 (m, 1H, aliphatic), 4.07–4.11 (m, 1H, aliphatic), 4.27–4.28 (m, 2H, aliphatic), 4.35–4.38 (m, 1H, aliphatic), 6.87–6.92 (d, 1H, *J* = 15 Hz), 7.00–7.15 (m, 2H, aromatic), 7.12–7.15 (m, 1H, aromatic), 7.22 (s, 1H), 7.27–7.29 (m, 1H, aromatic), 7.35–7.39 (m, 3H, aromatic), 7.45–7.50 (m, 3H, aromatic), 7.69–7.72 (d, 1H, *J* = 15 Hz), 7.81–7.82 (m, 1H, aromatic), 8.22–8.24 (m, 1H, aromatic); ^13^C-NMR (CDCl_3_, δ): 20.06, 43.27, 52.74, 68.33, 73.16, 106.95, 119.24, 119.37, 121.59, 121.87, 123.34, 124.09, 124.46, 126.02, 126.36, 126.65, 128.3, 130.06, 130.75, 133.95, 139.31, 140.54, 155.17, 156.43, 157.16,168.33; Elemental Analysis: Expected % (C_31_H_31_NO_4_): C, 77.31; H, 6.49; N, 2.91; Found % (C_31_H_31_NO_4_): C, 77.19; H, 6.66; N, 2.90.

#### 3.2.2. Synthesis of (*S*)-*N*-(2-hydroxy-3-(naphth-1-yloxy) propyl)-*N*-isopropyl-3-(3-phenoxyphenyl) acrylamide (***S*-2b**)

**Scheme 5:**
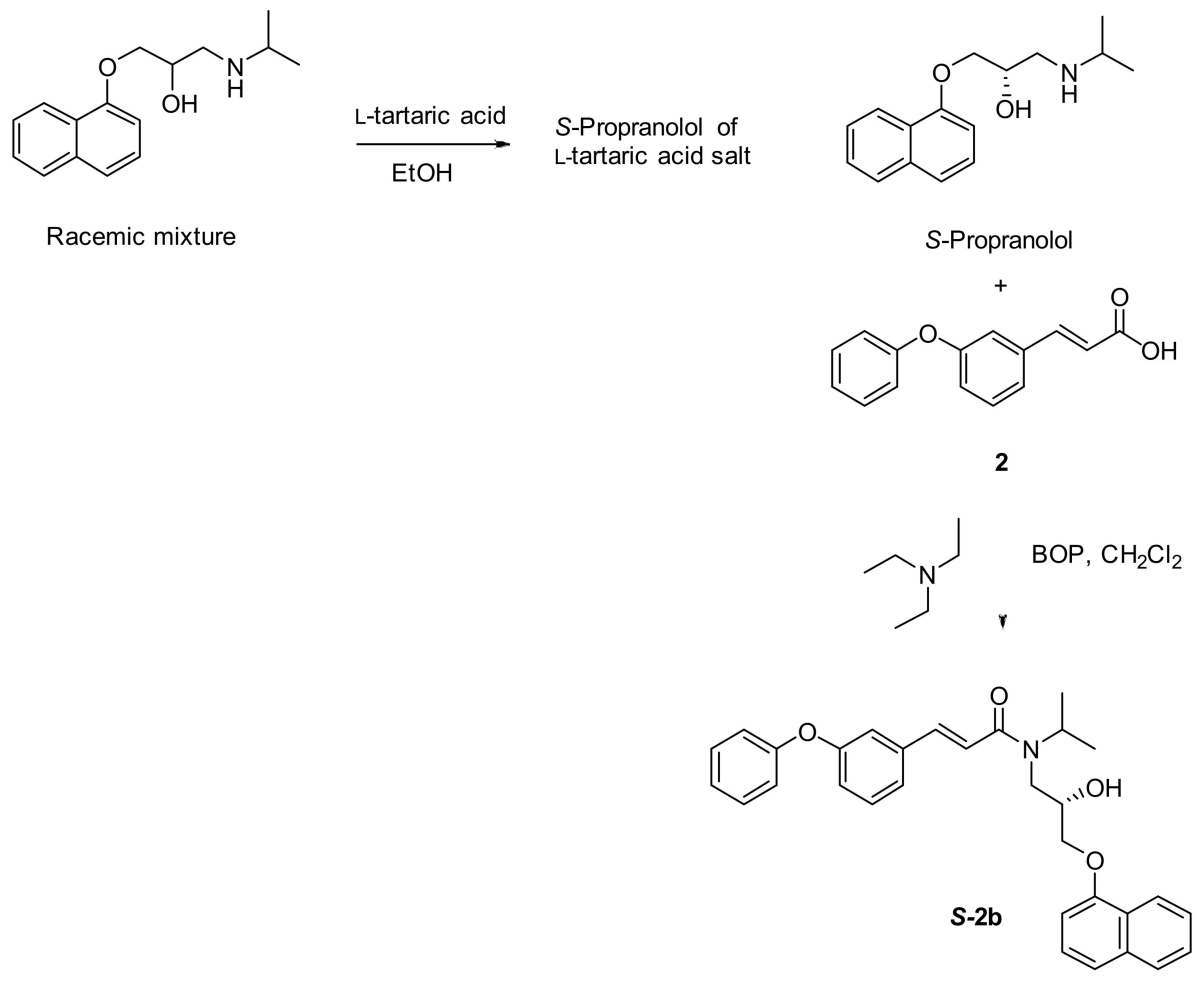
Synthesis and isolation of *(S)-N*-(2-hydroxy-3-(naphth-1-yloxy) propyl)-*N*-isopropyl-3-(3-phenoxy-phenyl) acrylamide (***S*-2b**).

A racemic mixture of propranolol (0.386 mmol) was dissolved in ethanol (1.2 mL) and l-tartaric acid (0.772 mmol) was added. The mixture was heated to reflux for 10 h and then allowed to cool to room temperature. After addition of diethyl ether into the cooled ethanol reaction mixture, a white solid was precipitated, filtered, collected, and dried. Then the precipitated solid was dissolved in water, neutralized using 10% solution of NaHCO_3_ followed by extractions from diethyl ether. The organic phase dried over Na_2_SO_4_ and after removal of organic solvent the desired 1:1 l-tartaric acid salt of *S*-propranolol weighed 0.18 g (88% yield). The l-tartaric acid salt was suspended in toluene (10 mL) and cooled with an ice water bath while a saturated solution of sodium bicarbonate (5 mL) was added dropwise. The temperature was maintained below a maximum of 25 °C. The clear, biphasic mixture was stirred for 20 min at 25 °C, and the layers were separated. The organic layer was washed with water (100 mL), dried over sodium sulfate and evaporated to provide a white crude solid of (*S*)-propranolol. Next (*E*)-3-(3-phenoxyphenyl)acrylic acid 2 (1 equivalent), (*S*)-propranolol (1.1 equivalents), BOP (1.2 equivalents) and triethylamine (4 equivalents) were dissolved in dry CH_2_Cl_2_ (5.0 mL) and stirred for 24 min at r.t., according to the previously reported general procedure (Scheme 2) to afford the title compound ***S*-2b** (Scheme 5) as a white solid.

*(E)-N-(3-(3-(2-amino-2-oxoethyl)phenoxy)-2-hydroxypropyl)-3-(3,4-dimethoxyphenyl)-N-isopropylacryl-amide* (**3a**). The crude product was crystalized by ethyl acetate. Yield: 80%; *R*_f_ (petroleum ether:ethyl acetate 1:1): 0.51; m.p.: 176–178 °C; IR (KBr, cm^−1^): 3358.3, 3298.3, 2937.5 1718.0, 1687.6, 1636.8; LC-MS (*m*/*z*): (C_25_H_32_N_2_O_6_) [M + 1] = 457.05; ^1^H-NMR (CDCl_3_, δ): 1.28–1.34 (m, 6H, -NH(CH_3_)_2_), 3.46–3.48 (m, 1H, aliphatic), 3.55 (m, 2H, aliphatic proton), 3.70–3.75 (m, 1H, aliphatic proton), 3.86–3.88 (m, 2H, aliphatic proton), 3.93 (s, 6H, OCH_3_ ), 4.08 (m, 1H, aliphatic proton), 4.35–4.38 (m, 1H, aliphatic), 5.71 (bs, 1H, -OH), 6.74–6.77 (d, 1H, *J* = 15 Hz), 6.86–6.92 (m, 3H, aromatic), 7.03 (m, 1H, aromatic), 7.13–7.20 (m ,3H, aromatic), 7.67–7.70 (d, 1H, *J* = 15 Hz); ^13^C-NMR (CDCl_3_, δ): 21.06, 42.36, 45.68, 51.18, 55.89, 56.02, 68.17, 70.62, 110.35, 112.87, 113.05, 114.03, 119.41, 122.18, 122.95, 129.65, 130.05, 135.36, 140.56, 151.62, 152.01, 157.92, 167.91, 172.52; Elemental Analysis: Expected % (C_25_H_32_N_2_O_6_): C, 65.77; H, 7.07; N, 6.14; Found % (C_25_H_32_N_2_O_6_): C, 65.47; H, 7.11; N, 6.38.

*(E)-3-(3,4-dimethoxyphenyl)-N-(2-hydroxy-3-(naphthalen-1-yloxy)propyl)-N-isopropylacrylamide* (**3b**). The crude product was purified by Prep TLC (ethyl acetate/petroleum ether 4:1)Yield: 62%; *R*_f_ (petroleum ether:ethyl acetate 1:1): 0.62; m.p.: 172–174 °C; IR (KBr, cm^−1^): 3369, 2962.4, 1718.5, 1636.8; LC-MS (*m*/*z*): (C_27_H_31_NO_5_) [M + 1] = 450; ^1^H-NMR (CDCl_3_, δ): 1.10 (s, 3H,-CH_3_), 1.13 (s, 3H,-CH_3_), 1.30–1.28 (m, 1H, aliphatic), 1.88–1.89 (m, 1H, aliphatic), 3.77–3.80 (m, 2H, aliphatic), 4.13–4.16 (m, 1H, aliphatic), 4.30 (bs, 1H, -OH), 6.68–6.78 (m, 3H, aromatic), 6.93–7.01 (m, 3H, aromatic), 7.22–7.24 (d, 1H, *J* = 15 Hz), 7.30–7.36 (m, 2H, aromatic), 7.58–7.61 (d, 1H, aromatic), 7.66–7.68 (m, 1H, aromatic), 8.10–8.12 (d, 1H, *J* = 15 Hz); ^13^C-NMR (CDCl_3_, δ): 21.51, 43.52, 50.85, 55.38, 55.56, 69.07, 71.32, 105.98, 111.03, 112.56, 118.52, 120.98, 121.73, 122.22, 124.5, 124.8, 124.91, 125.82, 126.26, 129.52, 134.47, 140.26, 150.02, 151.09, 154.09, 168.89; Elemental Analysis: Expected % (C_27_H_31_NO_5_): C, 72.14; H, 6.95; N, 3.12; Found % (C_27_H_31_NO_5_): C, 71.98; H, 7.25; N, 2.96.

#### 3.2.3. General Procedure for the One Pot Synthesis of Cinnamic Esters Derivatives **1c**–**d**

Meldrum’s acid (1 equivalent) was dissolved in toluene (50 mL), and then 1-adamantol or naphthalen-1-ol (1 equivalent) was added, respectively [31]. The mixture was heated and refluxed for 4 h. Then the reaction mixture was cooled to room temperature, followed by addition of benzaldehyde (0.2 equivalents), pyridine (2.5 mL) and piperidine (0.25 mL). The stirring continued at room temperature for 24 h, TLC monitoring until the reaction was completely finished. The solvents were distilled out under vacuum; the residue was dissolved in diethyl ether (30 mL), washed with saturated sodium bicarbonate solution, diluted with aqueous 1M hydrochloric acid solution and water. The ether phase was dried by anhydrous MgSO_4_ overnight. After removal of the drying agent, the solvent was distilled out to afford a crude solid which was recrystallized from a mixture of dichloromethane (DCM) and diethyl ether.

*(E)-adamantan-1-yl cinnamate* (**1c**). Yield: 32%; *R*_f_ (petroleum ether:ethyl acetate 1:1): 0.71; m.p.: 183–185 °C; IR (KBr, cm^−1^): 2921.5, 2852.1, 1711.4, 1687.6, 1654; LC-MS (*m*/*z*): (C_19_H_22_O_2_) [M + 1] = 283.05, [M + CH_3_CN + MeOH] = 355, [M + CH_3_CN + MeOH + K] = 395; ^1^H-NMR (DMSO, δ): 1.59–1.68 (m, 6H, aliphatic), 1.73–1.74 (m, 6H, aliphatic), 2.15–2.17 (m, 3H, aliphatic proton),7.17–7.20 (m, 4H, aromatic), 7.25–7.29 (m, 3H, aromatic); ^13^C-NMR (DMSO, δ): 33.74, 34.75, 45.68, 78.84, 118.56, 128.86, 128.98, 129.31, 132.40, 143.65, 167.31; Elemental Analysis: Expected % (C_19_H_22_O_2_): C, 80.82; H, 7.85; Found % (C_19_H_22_O_2_): C, 80.68; H, 7.55.

*(E)-naphthalen-1-yl cinnamate* (**1d**). Yield: 52%; *R*_f_ (petroleum ether:ethyl acetate 1:1): 0.65; m.p.: 210–212 °C; IR (KBr, cm^−1^): 2869.6, 2361.4, 1710.1, 1652.8; LC-MS (*m*/*z*): (C_19_H_14_O_2_) [M + Na] = 297; ^1^H-NMR (CDCl_3_, δ): 6.83–6.80 (d, 1H, *J* = 15 Hz), 7.33–7.35 (d, 1H, *J* = 10 Hz), 7.39–7.40 (m, 1H, aromatic), 7.45–7.47 (m ,2H, aromatic), 7.51–7.53 (m ,3H, aromatic), 7.65–7.66 (m, 2H, aromatic), 7.77–7.79 (d, 1H, *J* = 10 Hz), 7.89–7.95 (m, 2H, aromatic), 7.99–8.02 (d, 1H, *J* = 15 Hz); ^13^C-NMR (DMSO, δ): 114.18, 115.72, 120.89, 122.63, 125.13, 126.10, 126.32, 126.67, 128.86, 129.21, 130.12, 131.56, 134.15, 147.59, 147.85, 167.23; Elemental Analysis: Expected % (C_19_H_14_O_2_): C, 83.19; H, 5.14; Found % (C_19_H_22_O_2_): C, 83.34; H, 4.99.

#### 3.2.4. General Procedure for the One Pot Synthesis of Cinnamic Esters Derivatives **1e**–**f**

To a stirred solution of cinnamic acid **1** (1 equivalent) and 2-(benzylamino) ethan-1-ol (1.1 equivalents) in DCM (25 mL), BOP (1.2 equivalents) and triethylamine (4 equivalents) were added [30]. The reaction mixture was stirred until the reaction stopped (TLC monitoring) after 24 h stirring at room temperature, then the DCM was removed under reduced pressure and ethyl acetate (80 mL) was added to the residue. The resulting solution was washed with 10% citric acid (2 × 20 mL), 10% solution of NaHCO_3_ (3 × 20 mL), water (20 mL) and brine (2 × 20 mL), dried over anhydrous sodium sulfate, filtered and concentrated under reduced pressure. These compounds purified chromatographically (Prep TLC, ethyl acetate/petroleum ether 4:1).

*(E)-2-(benzylamino) ethyl 3-phenylacrylate* (**1e**). The reaction mixture was treated according to the general procedure (Scheme 3), to give the titled compound **1e** as a semisolid after Prep TLC chromatography using mixture of ethyl acetate/petroleum ether 4:1. Yield: 58%; *R*_f_ (MeOH:H_2_O 7:3): 0.76; LC-MS (*m*/*z*): (C_17_H_17_NO_2_) [M + 1] = 268; ^1^H-NMR (CDCl_3_, δ): 3.15 (s, 1H, -NH), 3.63–3.67 (m, 2H, aliphatic), 3.77–3.83 (m, 2H, aliphatic), 4.77–4.84 (m, 2H, aliphatic), 6.86–6.89 (d, 1H, *J* = 15 Hz), 7.24–7.25 (m, 1H, aromatic), 7.30–7.55 (m, 9H, aromatic), 7.80–7.83 (d, 1H, *J* = 15 Hz); Elemental Analysis: Expected % (C_17_H_17_NO_2_): C, 76.38; H, 6.41; N, 5.24; Found % (C_17_H_17_NO_2_): C, 76.28; H, 6.19; N, 5.01.

*2-(N-benzyl cinnamamido) ethyl cinnamate* (**1f**). The reaction mixture was treated according to the general procedure (Scheme 3), previously reported, to give the titled compound **1e** as a semisolid after Prep TLC chromatography using mixture of ethyl acetate/petroleum ether 4:1. Yield: 82%; *R*_f_ (MeOH:H_2_O 7:3): 0.43; LC-MS (*m*/*z*): (C_27_H_25_NO_3_) [M + 1] = 412; ^1^H-NMR (CDCl_3_, δ): ^1^H-NMR (CDCl_3_, δ): 3.75 (t, 1H, *J* = 10 Hz), 3.83 (t, 1H, *J* = 10 Hz), 4.32 (t, 1H, *J* = 10 Hz), 4.45 (t, 1H, *J* = 10 Hz), 4.81 (s, 2H, aliphatic), 6.29–6.37 (m, 1H), 6.81–6.84 (d, 1H, *J* = 15 Hz), 7.09–7.12 (d, 1H, *J* = 15 Hz), 7.24–7.25 (m, 1H), 7.30–7.66 (m, 14H, aromatic), 7.76–7.83 (m, 1H, aromatic); Elemental Analysis: Expected % (C_27_H_25_NO_3_): C, 78.81; H, 6.12; N, 3.40; Found % (C_27_H_25_NO_3_): C, 78.68; H, 6.49; N, 3.24.

### 3.3. Physicochemical Studies

#### Determination of *R*_M_ Values

Reversed phase TLC (RP-TLC) was performed on silica gel plates impregnated with 5% (*v*/*v*) liquid paraffin in light petroleum ether. The mobile phase was a methanol/water mixture (70/30, *v*/*v*). The plates were developed in closed chromatography tanks saturated with the mobile phase at 24 °C. Spots were detected under UV light. *R*_M_ values were determined from the corresponding *R*_f_ values (from five individual measurements) using the equation *R*_M_ = log[(1/*R*_f_) − 1] (Table 1) [46].

### 3.4. Biological In Vitro Assays

Each in vitro experiment was performed at least in triplicate and the standard deviation of absorbance was less than 10% of the mean. For the in vitro assays, a stock solution (1% DMSO in the appropriate buffer with the tested compound diluted under sonication) was prepared from which several dilutions were made with the appropriate buffer.

#### 3.4.1. Inhibition of Linoleic Acid Lipid Peroxidation

Production of conjugated diene hydro peroxide by oxidation of sodium linoleate in an aqueous solution was monitored at 234 nm. AAPH was used as a free radical initiator. Ten microliters of the 16 mM sodium linoleate solution was added to the UV cuvette containing 930 μL of 0.05 M phosphate buffer, pH 7.4 prethermostated at 37 °C. The oxidation reaction was initiated at 37 °C under air by the addition of 50 μL of 40 mM AAPH solution. 10 μL of the appropriate solutions of the tested compounds were added in the mixture. Lipid oxidation was measured in the presence of the same level of DMSO. The rate of oxidation at 37 °C was monitored by recording the increase in absorption at 234 nm caused by conjugated diene hydro peroxides [24]. The results were compared to the appropriate standard inhibitor Trolox (93%) (Table 1).

#### 3.4.2. Soybean Lipoxygenase Inhibition Study In Vitro

In vitro study was evaluated as reported previously [24]. The tested compounds were dissolved in DMSO (10 μL) and incubated at room temperature with sodium linoleate (100 mM) and 0.2 mL of enzyme solution (1/9 × 10^−4^ *w*/*v* in saline) in buffer pH 9 (tris) at room temperature (final volume 1 mL). The conversion of sodium linoleate to 13-hydroperoxylinoleic acid at 234 nm was recorded and compared with the appropriate standard inhibitor NDGA (IC_50_ = 0.45 μM). Several concentrations were used for the determination of IC_50_ values. The results are given in Table 1 expressed as IC_50_ values or % inhibition at 100 μΜ.

#### 3.4.3. Inhibition of Trypsin Induced Proteolysis In Vitro

The test was carried out according to a modified Kunitz method. Bovine albumin was used as substrate for trypsin [24]. The reaction mixture consisted of 200 μL of 0.075 mg/mL trypsin and 780 μL of phosphate buffer pH 7.6 (including the tested compounds (20 μL in DMSO, final concentration 100 μM) preincubated at 37 °C for 20 min and then 1 mL of albumin (stock solution 6 g/100 mL in phosphate buffer 0.1 M, pH 7.6) was added and incubated at 37 °C for 30 min. After the incubation 1 mL of 5% trichloroacetic acid was added to the incubated solution to stop the enzyme’s reaction and allowed to set at room temperature for 1 h. Then the solution was filtered and the absorption of the filtered solution was measured at 280 nm. Salicylic acid was used as a reference compound (Table 1).

#### 3.4.4. Evaluation of the Cytotoxicity

L929 mouse fibroblasts cells were cultured in EMEM supplemented with 10% horse serum, 2 mM l-glutamine, 1 mM sodium pyruvate, 0.1 mM nonessential amino acids, 1.5 g/L sodium bicarbonate and 100 μg/mL penicillin–streptomycin at 37 °C in a humidified atmosphere with 5% CO_2_. L929 cells were plated into 24-well plates at a density 5104 cells/well and allowed to attach and grow for 24 h. The supernatant in each well then replaced with medium containing various concentrations (1, 10, 20, 50 and 100 μM) of compounds **1a**–**b**, **2a**–**b**, atenolol, propranolol, acids **1** and **2**, which presented more interesting results in the in vitro experiments. After 24 h incubation, the supernatant was removed and the cells were washed with PBS. The cells were detached with 0.25% trypsin, transferred to FACS tubes and then centrifuged (1600 rpm for 5 min) and the pellet washed with PBS. After washing, the cells in the pellet were incubated with 5 μL propidium iodide (PI) solution (1 mg/mL) for 1 min [44]. The PI fluorescence (cell death) was determined with flow cytometry, FACS Calibur, Coulter Epics XL-MCL (Beckman, Inc., Mount Holly, NJ, USA). The analysis of flow cytometry data was performed with WinMDI analysis program.

### 3.5. Computational Methods, Docking Simulations

All the molecules were constructed with the ChemDraw program [47] and converted into 3D-Structures with the OpenBabel program [48] by using MMFF94 force field. Protein setup was performed using the UCSF Chimera software [49,50] and to generate docking input files and to analyze docking results. Docking was carried out with an exhaustiveness value of 10 and a maximum output of 20 docking modes. The AnteChamber PYthon Parser interface (ACPYPE) tool [51] was employed to generate the topologies of the ligands. ACPYPE tool is written in python to use Antechamber [52,53] to generate topologies for chemical compounds was used for the parametrization of the ligands. Energy minimizations where carried out with the molecular simulation toolkit GROMACS [54] using the AMBER99SB-ILDN force field [55]. Docking calculations were performed with the software AutoDock Vina [56]. The PyRx program [57] was employed to generate the docking input files and to analyze the docking results. The proteins were considered rigid.

#### Molecular Docking Studies on Soybean Lipoxygenase

For the docking studies, we used soybean lipoxygenase enzyme (the 1RRH soybean lipoxygenase) because of its availability and its highly characterized structure [58], available from the Protein Data Bank (PDB) with a resolution of 2 Å [59]. Docking studies were performed for hybrid **2b** due to its overall significant biological profile in correlation with its in vitro results.

Supplementary material with the docking studies of the novel compounds as images, is provided (Figure S1).

## 4. Conclusions

The present study shows that the synthesized CA derivatives represent a promising class of multitarget compounds influencing several biological targets, in our case lipoxygenase and trypsin as well anti-lipid peroxidation activity. The amide **1a** was found to be the most potent anti-proteolytic agent. Racemic mixture **2b** prepared from phenyloxyphenyl cinnamic acid and propranolol showed high anti-lipid peroxidation activity (84%) in combination with high anti-LOX and trypsin inhibitory activity. The *S* isomer of **2b** presented also an interesting multitarget biological profile in vitro. The esters were found less active than the amides. The majority of the derivatives are quite potent lipid peroxidation inhibitors. Allosteric interactions might govern the LOX-inhibitor binding. Compounds **3a** and **3b**, derived from methoxylated caffeic acid, present a promising combination of in vitro inhibitory and anti-oxidative activities.

Polypharmacology remains one of the major challenges in drug development, and it opens novel avenues to rationally design next generation of more effective agents. Thus, the designed molecules could be successfully used as new multifunctional agents. Further investigations are in progress: (1) to discover any vasoconstriction and hypertension improvement, the cardioprotective profile, the regulation of the heart rate, the decrease of contraction and the reduction of the oxygen requirements of heart muscle induced by compounds **1a** and **2b** in vivo; and (2) to study the in vivo metabolic behavior and to define if the amides as well as the esters are hybrids or not.

## Figures and Tables

**Table 1:** Lipophilicity (*R*_M_ values), % Inhibition of lipid peroxidation (ILP%), in vitro inhibition of soybean lipoxygenase (IC_50_ μΜ or LOX Inh. %) and in vitro inhibition of trypsin induced proteolysis (IC_50_ μΜ or Trypsin Inh. %) by cinnamic derivatives.

Compounds	*R*_M_^a^ (±SD) ^b^	ILP% @100 μΜ ^b,c^	IC_50_ μΜ or LOX Inh. % @ 100 μΜ ^b,c^	IC_50_ μΜ or Trypsin Inh. % @100 μM ^b,c^
**1 ^d^**	−0.485 ± 0.044	78	56 μM	55 μM
**2 ^d^**	−0.41 ± 0.016	84	66 μM	na
**3 ^d^**	−0.869 ± 0.0614	86	na	na
**1a**	−0.720 ± 0.012	97	66 μΜ	0.315 μΜ
**1b**	0.411 ± 0.061	69	50 μΜ	10 μΜ
**2a**	0.128 ± 0.0204	86	35 μΜ	0.325 μΜ
**2b**	0.907 ± 0.008	84	6 μΜ	0.425 μΜ
**3a**	−0.841 ± 0.0014	86	10 μΜ	1 μΜ
**3b**	0.0819 ± 0.01	71	10 μΜ	0.516 μΜ
**1c**	0.911 ± 0.0112	20	na	49 μΜ
**1d**	0.0889 ± 0.002	na	27.5 μΜ	na
**1e**	−0.374 ± 0.0176	60	100 μΜ	na
**1f**	0.1402 ± 0.0227	na	20%	100 μΜ
***S*-2b**		86	6 μM	2.75 μM
NDGA			0.45 μM	
Trolox		93		
Atenolol		na	22.5 μΜ	na
Propranolol		na	42 μΜ	na
Salicylic acid				53.6%

na: no activity under the reported experimental conditions. Means within each column differ significantly (*p* < 0.05); @: at; ^a^ *R*_M_ values are the average of at least 5 measurements [MeOH:H_2_O (70/30)]; ^b^ SD standard deviation; ^c^ Values are means (±SD < 10%) of three or four different determinations; ^d^ Values are referred to cinnamic acids; IC_50_ represents the concentration of the tested compounds that is required for 50% inhibition in vitro.

## Supplementary Materials

Supplementary Materials are available online, Figure S1: Docking studies of the novel compounds.

## Abbreviations

AAPH	2,2-azobis (2-amidinopropane) dihydrochloride
ACE	Angiotensin converting enzyme
ACPYPE	AnteChamber PYthon Parser interface
BOP	*O*-(benzotriazol-1-yl)-*N*,*N*,*N*′,*N*″-tetramethyluronium hexafluorophosphate
CA	cinnamic acid
LOX	Lipoxygenase
NDGA	nordihydroguaiaretic acid
Prep TLC	Preparative Thin Layer Chromatography
RPTLC	Reverse-phase thin layer chromatography
