# Supplementary material for: Multifunctional Cinnamic Acid Derivatives
Source: Molecules. 2017 Jul 25;22(8):1247. doi: 10.3390/molecules22081247 (PMC6152057; doi:10.3390/molecules22081247)
Supplement: Supplementary file 1 [file molecules-22-01247-s001.pdf]

Multifunctional Cinnamic Acid Derivatives

Aikaterini Peperidou [1], Eleni Pontiki [1], Dimitra Hadjipavlou-Litina [1,*], Efstathia Voulgari [2] and Konstantinos Avgoustakis [2]

[1] Department of Pharmaceutical Chemistry, School of Pharmacy, Faculty of Health Sciences, Aristotle University of Thessaloniki, Thessaloniki 54124, Greece; katerina.peperidou@gmail.com (A.P.); epontiki@pharm.auth.gr (E.P.)

[2] Department of Pharmaceutical Technology and Pharmaceutical Analysis, School of Pharmacy, University of Patras, Rio Patras 26504, Greece; efiv48@hotmail.com (E.V.); avgoust@upatras.gr (K.A.)

* Correspondence: hadjipav@pharm.auth.gr; Tel.: +30-231-099-7627; Fax: +30-231-099-7679

Figure S1. Docking studies of the synthesized novel derivatives.

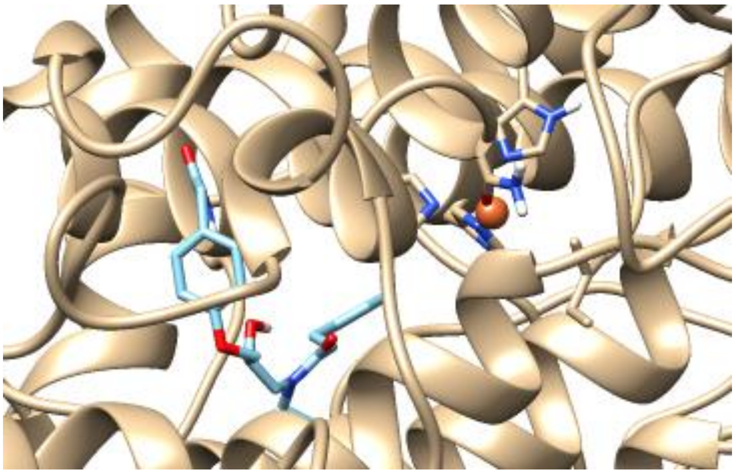

1a

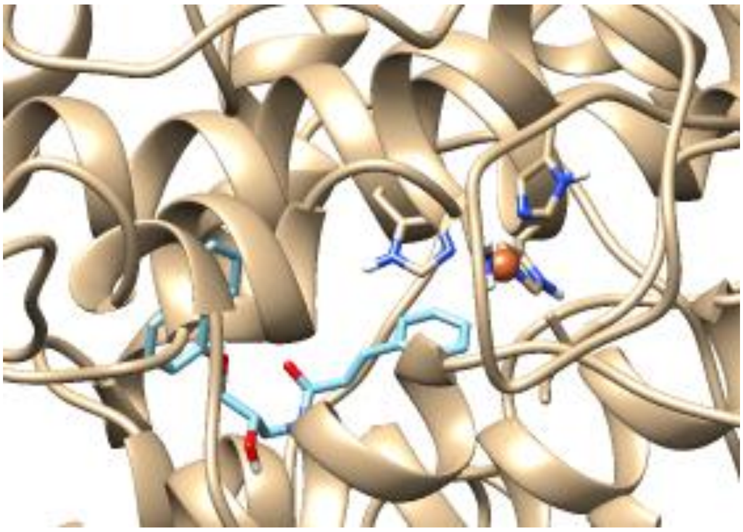

1b

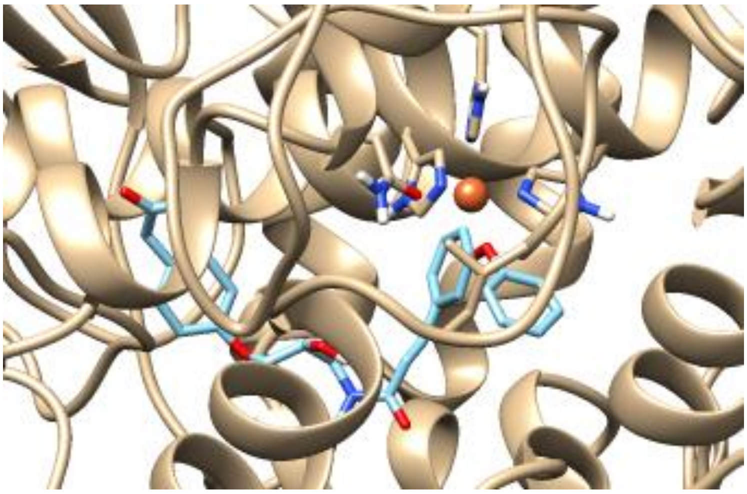

2a

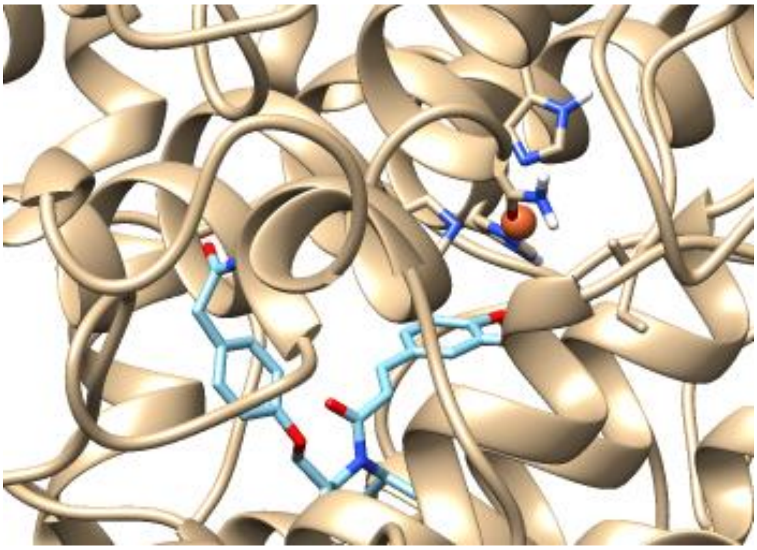

3a

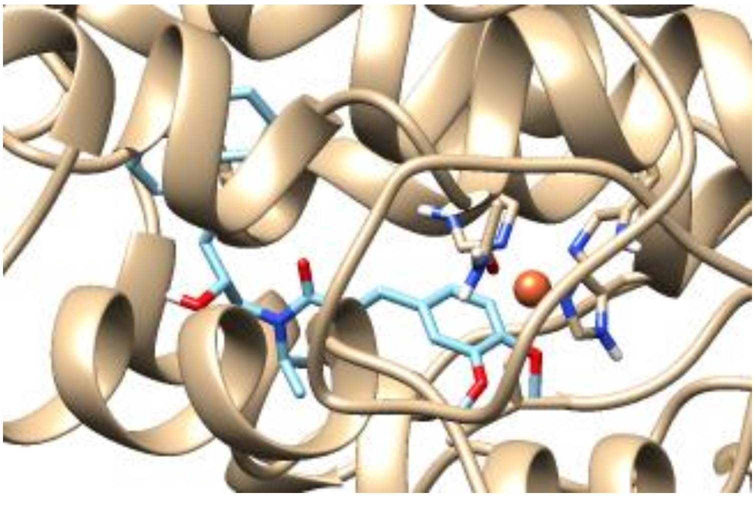

3b

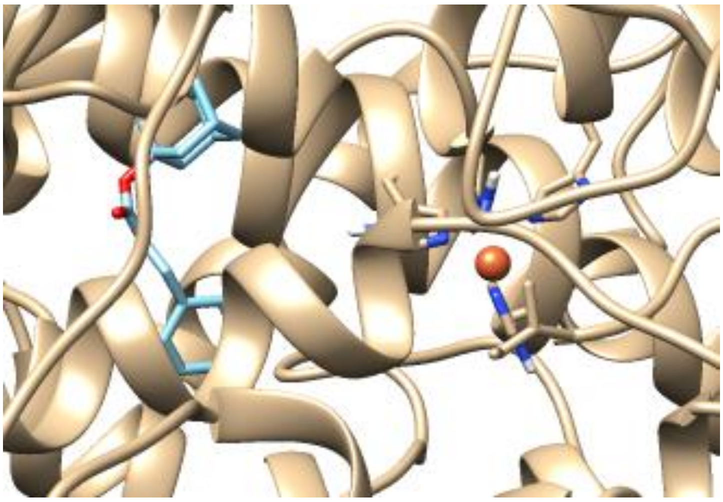

1c

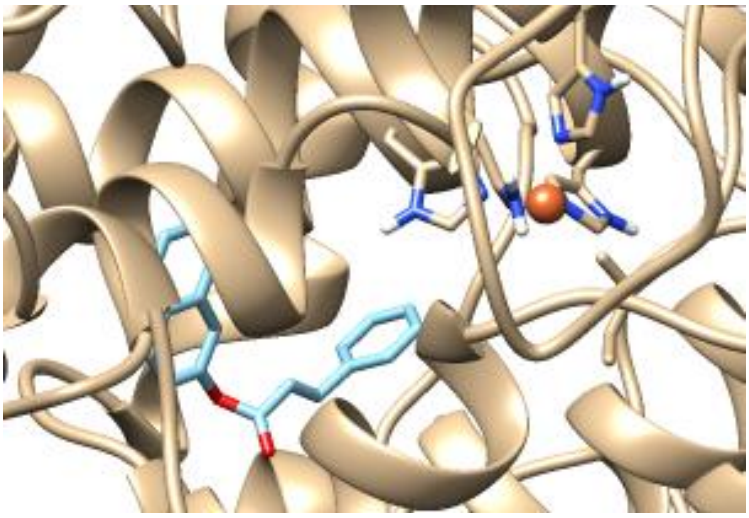

1d

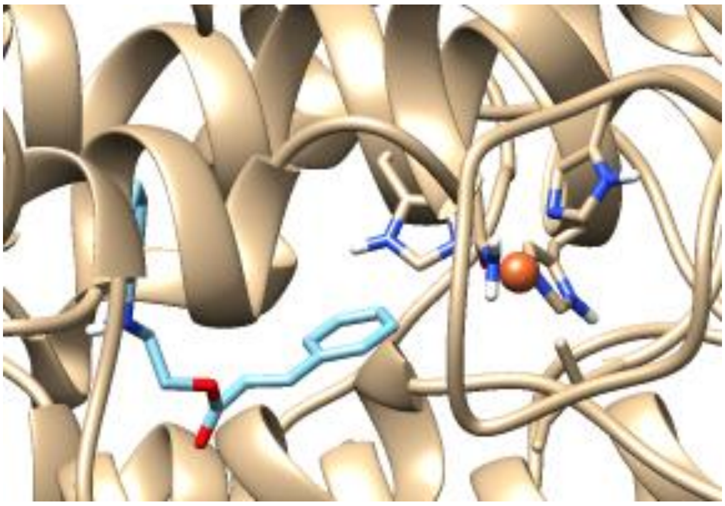

1e

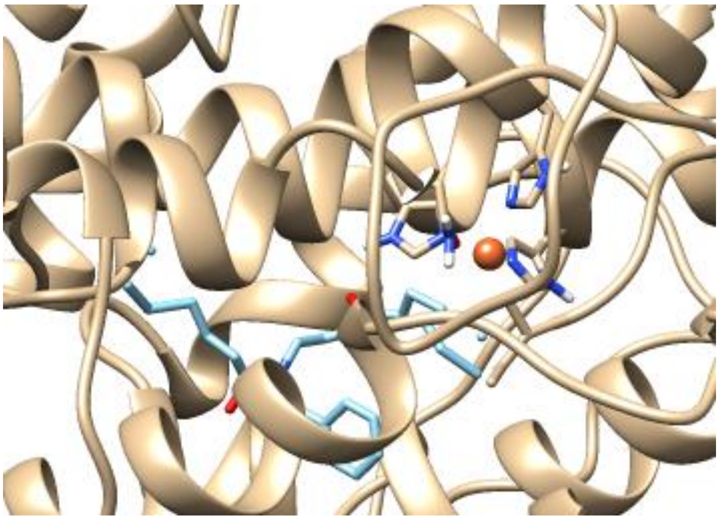

1f
